# The influence of social media and cultural ideals on body dysmorphic disorder among adult males in the UAE

**DOI:** 10.3389/fpsyt.2025.1613623

**Published:** 2025-07-18

**Authors:** Kholod Haj Hussain, Abdul Rahman Al Midani, Mohamad Abdallah, Aisha Alyassi, Haya Alzubaidy, Shahad Alrashed, Jibran Sualeh Muhammad, Hamid A. Alhaj

**Affiliations:** ^1^ College of Medicine, University of Sharjah, Sharjah, United Arab Emirates; ^2^ Queen Square Institute of Neurology, University College London, London, United Kingdom; ^3^ Department of Biomedical Sciences, College of Medicine and Health, University of Birmingham, Birmingham, United Kingdom

**Keywords:** body dysmorphic disorder, social media, cosmetic surgery, muscle dysmorphia, males

## Abstract

**Introduction:**

Body Dysmorphic Disorder (BDD) is a psychiatric condition characterized by an excessive preoccupation with perceived flaws in physical appearance, often resulting in significant emotional distress and impaired functioning. Although social influences are believed to contribute to the development of BDD, their specific impact remains underexplored, especially among males in the Middle East. This study aims to investigate the relationship between BDD symptoms and social factors, with a particular emphasis on the role of social media among adult males in the United Arab Emirates (UAE).

**Methods:**

A cross-sectional survey was conducted among men using a self-administered 30-item questionnaire. The instrument covered demographics, the Body Dysmorphic Disorder Questionnaire (BDDQ), body image and cosmetic surgery attitudes, social media use, and exercise behaviors. The questionnaire was translated using a forward-backward translation technique and reviewed for clarity and cultural appropriateness in accordance with WHO guidelines. Bivariate analyses were conducted to explore associations between variables. Chi-square and Fisher’s exact tests were used for categorical variables, and t-tests for continuous variables, with statistical significance set at *p* < 0.05.

**Results:**

Of the 403 participants, 53 (13.2%) met the BDDQ cutoff criteria, and 15 (3.7%) screened positive for BDD (excluding weight-related concerns). A significant association was found between BDD screening positivity and perceived negative impact of social media (χ²(2) = 19.92, *p* <.001). Similarly, perceived cultural pressure to attain an ideal appearance was significantly associated with higher BDDQ positivity. Participants who screened positive for BDD were significantly more likely to report physical discomfort (OR = 4.9, 95% CI: [1.5, 15.8], *p* = 0.005), concern about others’ perceptions of their bodies (OR = 3.9, 95% CI: [1.2, 12.3], *p* = 0.017), and interest in cosmetic surgery (OR = 5.8, 95% CI: [2.0, 16.4], *p* = 0.002). Multivariate analysis showed that perceived negative social media impact, specific cosmetic concerns, and lower BMI were independent predictors of BDD.

**Conclusions:**

This study highlights the significant role of social media, cultural appearance pressures, and attitudes toward cosmetic surgery in the manifestation of BDD symptoms among males in the UAE. Culturally sensitive public health initiatives should focus on promoting healthy body image and increasing awareness of BDD.

## 1 Introduction

### 1.1 Background

Body Dysmorphic Disorder (BDD) is a mental health condition characterized by an obsessive preoccupation with personally perceived flaws in one’s physical appearance, often so negligible or unnoticed by others, leading to profound distress and significant impairment in quality of life (1, 2). Individuals suffering from BDD commonly experience significant impairments in both social and occupational functioning, resulting in consequences such as social withdrawal, contemplation of suicide, and substance misuse (3). Classified under obsessive-compulsive and related disorders in the Diagnostic and Statistical Manual of Mental Disorders, 5th edition (DSM-5), BDD diagnosis hinges on a marked preoccupation with physical flaws and repetitive behaviors, such as body checking, seeking validation, grooming rituals, comparing appearances, rigorous exercising, and contemplating cosmetic surgeries, all aimed at addressing these concerns (4, 5). Without proper intervention, BDD may result in persistent absenteeism, diminished productivity, unemployment, and marital discord (6–8).

BDD is recognized as a relatively prevalent psychiatric disorder, with its prevalence varying significantly across different settings, countries, age groups, occupations, and genders. It has been suggested that BDD is underdiagnosed, especially in men, in whom symptoms may be mistaken for general insecurity or obsessive gym habits. Studies have shown that in the general population, prevalence rates range from 0.7% to 2.9% (9–11), with this number increasing in certain subpopulations (12). Overall, BDD has a weighted global prevalence of 1.9% (13).

The development and consequences of BDD exhibit notable variations between men and women. While women may face intense societal emphasis on beauty, thinness, and flawless skin, males are more likely to be influenced by ideals around muscularity, strength, and height. In women, significant risk factors for BDD include socioeconomic status and a history of domestic violence (14). Conversely, among men, this condition has a more pronounced impact on individuals living in solitary conditions, with height emerging as a significant source of dissatisfaction with physical appearance (15).

The interplay between BDD and sociocultural influences has been increasingly recognized, particularly in relation to exercise addiction and the pervasive impact of media. Individuals with BDD may engage in compulsive exercise behaviors as a means of modifying or controlling their appearance, reinforcing maladaptive patterns that contribute to psychological distress. BDD often manifests in men as muscular dysmorphia, characterized by an excessive preoccupation with physical appearance and a persistent sense of muscular inadequacy, which may result in the development of exercise addiction and inappropriate supplement use (16–18).

This study’s theoretical underpinning is provided by the tripartite impact model, which holds that peers, family, and the media all work together to create body image through internalization of appearance standards and appearance comparison (19). The internalization of appearance standards and people’s propensity to compare their appearances are caused by these forces taken together. This model offers a strong framework for comprehending how multiple factors might combine to impact behaviors and perceptions of one’s body, especially when it comes to cosmetic surgery concerns. Similarly, a critical factor in the exacerbation of BDD symptoms is the influence of media, especially social media, which serves as a primary platform for the dissemination of idealized body standards (20). The widespread portrayal of unrealistic and digitally altered physiques fosters heightened self-scrutiny and dissatisfaction among individuals vulnerable to body image concerns (21–23). Studies suggest that excessive exposure to curated and filtered images on social networking sites is associated with increased body dissatisfaction, compulsive comparison behaviors, and a greater likelihood of engaging in appearance-altering practices, including excessive exercise.

The role of social media in shaping body image perceptions is particularly salient in the digital age, where platforms such as Instagram, TikTok, and Facebook perpetuate aesthetic norms that may not be attainable for the general population. This constant exposure to idealized body representations can reinforce negative self-evaluations and contribute to the development or exacerbation of BDD symptoms. Furthermore, the interactive nature of social media allows for immediate feedback in the form of likes, comments, and shares, which can reinforce individuals’ concerns about their appearance and encourage compulsive behaviors, including excessive exercise as a means of achieving perceived societal standards. Social media celebrity culture has a considerable influence on the development of these issues and the pursuit of cosmetic surgery (24, 25), which often translates into seeking cosmetic surgeries in an attempt to resolve their body image issues (21–23).

Studies conducted in the Middle East have emphasized the widespread and early influence of conventional and social media on body image. Investigations into Arab youth reveal that social media platforms are central arenas where individuals engage with and feel pressure to meet evolving beauty standards, often prioritizing appearance and self-presentation (26). A research studies of Iraqi women showed that while people balance traditional and contemporary aesthetic norms, exposure to idealized beauty standards on social media can increase their desire for cosmetic treatments (27). Conversely, Alshaalan et al. provided examples of how social media specifically affects Saudi Arabian consumers’ decisions regarding periocular cosmetic implants. According to this research, prospective patients are using social media accounts more frequently to research procedures, which affects how they view them and whether or not they decide to have such surgeries (28).

The United Arab Emirates (UAE) has a unique atmosphere where traditional ideas of masculinity and international standards of beauty collide due to rapid industrialization, more exposure to Western media, and changing cultural norms. Despite these significant sociocultural shifts, little is known about how BDD and body image issues impact men in the UAE, as the majority of studies have been on women. Although adult males in UAE may have unique and changing needs concerning muscularity, fitness, and appearance, little is known about their experiences.

This study is one of the few to concentrate solely on adult males, a demographic that is underrepresented in regional body image and BDD research, in contrast to earlier studies. To capture the distinct sociocultural elements influencing body image and BDD in this population, it makes use of a culturally specific, piloted, and translated questionnaire. Additionally, the study provides a thorough examination of the variables influencing BDD symptoms by integrating measurements of social media use, exercise, body image, and cosmetic attitudes. The results offer fresh insights into the ways that media exposure, modernization, and traditional values combine to influence men’s perceptions of their bodies in the UAE.

### 1.2 Aims and hypothesis

This study aimed to investigate the associations between body dysmorphic disorder symptoms and (1) social media usage, (2) attitudes toward cosmetic procedures, (3) exercise behaviors, and (4) perceived cultural pressures among adult males in the UAE.

Our hypothesis was that among adult males in the UAE, there would be a significant correlation between increased BDD symptoms and higher levels of social media use, more positive attitudes toward cosmetic procedures, increased exercise, and stronger perceived cultural pressure to achieve an ideal appearance.

By concentrating on men, the study fills a gap in the regional literature and addresses recent findings of increased interest in cosmetic surgery and BDD prevalence among young men in the Middle East.

## 2 Methodology

### 2.1 Study design

Participants were recruited for this online, cross-sectional study using social media platforms. A piloted, self-reported questionnaire with 30 items divided into four sections - demographics, BDDQ, body image and cosmetics, and exercise and social media - was used to collect the data. Informed consent was obtained from all participants prior to their involvement in the study. Ethical approval was granted by the Research Ethics Committee at the University of Sharjah (REC-21-02-10-04-S), and the study was conducted in accordance with the principles outlined in the Declaration of Helsinki.

### 2.2 Sample size and sampling procedures

Convenience sampling was used to recruit male participants aged 18 and above residing in the United Arab Emirates (UAE), proficient in either English or Arabic, and with access to social media platforms. The questionnaire was distributed through social media platforms, including WhatsApp, Instagram, and Twitter. The minimum sample size (n=385) was calculated using the formula n = (z² * p * (1 - p))/e²), where Z = 1.96 (95% CI), p = 0.5 (maximum variability), and e = 0.05 (margin of error) (29). This calculation ensures adequate statistical power for prevalence estimation.

### 2.3 Questionnaire

Demographics, the official Body Dysmorphic Disorder Questionnaire (BDDQ), a section on body image and cosmetics, and a section on exercise and social media comprised the four primary components of the questionnaire. Nine closed-ended questions make up the BDDQ, a validated screening instrument based on DSM-IV criteria (see **Supplementary Table 1**). Only those who answered “yes” to the first two questions proceeded to the rest. A score of four or above indicates possible BDD and calls for additional testing.

Additional items were created by the research team for the sections on body image, exercise, cosmetic attitudes, and social media in order to investigate attitudes and behaviors pertinent to the study population. These non-BDDQ items were not modified from other standardized or validated instruments, and instead were simple, closed-ended questions such as multiple choice or yes/no as opposed to Likert-type scales. There were five of these items in the section on body image and cosmetics (see **Supplementary Table 2**), and eight in the section on exercise and social media (see **Supplementary Table 3**).

The WHO-recommended forward and back-translation approach was used to translate the questionnaire into Arabic. The English version was independently translated into Arabic by two multilingual specialists, and the back-translation was done by a third specialist. Consensus was used to settle disagreements. Versions in both languages were tested for suitability and clarity. Cultural pressure was assessed using two items in the exercise social media section: “Have you ever felt pressured to fit into a culturally “ideal” look (e.g., strong and muscular for males)?” and “Do you believe that social media has negatively impacted your feelings about your appearance?”

### 2.4 Pilot study

A pilot test involving 15 participants was conducted to assess the questionnaire’s clarity and completion time. Participants were selected to represent the desired demographic. The results of the pilot test were utilized to refine the questionnaire and were not included in the final analysis. No formal psychometric validation was performed due to the single-item or simple nature of most non-BDDQ questions and the small pilot sample size.

### 2.5 Statistical analysis

The data collected in this study was analyzed using the Statistical Package for Social Sciences (SPSS) version 25. Descriptive statistics, including frequencies, percentages, and mean ± standard deviation (SD), were utilized to summarize the data. Bivariate analysis was performed to examine the relationships between variables. Categorical variables were analyzed using the Chi-square test or Fisher’s exact test, as appropriate. Continuous variables were assessed using the independent t-test. Normality was evaluated using the Shapiro-Wilk test, and in case of non-normal distribution, non-parametric alternatives (such as Mann–Whitney U test) were used. A significance level of p < 0.05 was employed to determine statistical significance. A multivariate logistic regression analysis to identify independent variables associate with body dysmorphic disorder in UAE men was performed. The model’s variables were chosen for their clinical significance. The presence of body dysmorphic disorder was the dependent variable, and the independent variables were body image-related (body image cosmetic concerns, social media impact, social pressure regarding ideal appearance, perceived insufficient muscularity, and use of multiple clothing layers), behavioral (steroid use, gym use, exercise frequency, and social media usage), and demographic (age group, BMI, marital status, ethnicity, and average income).

## 3 Results

### 3.1 Demographics

**Figure 1:**
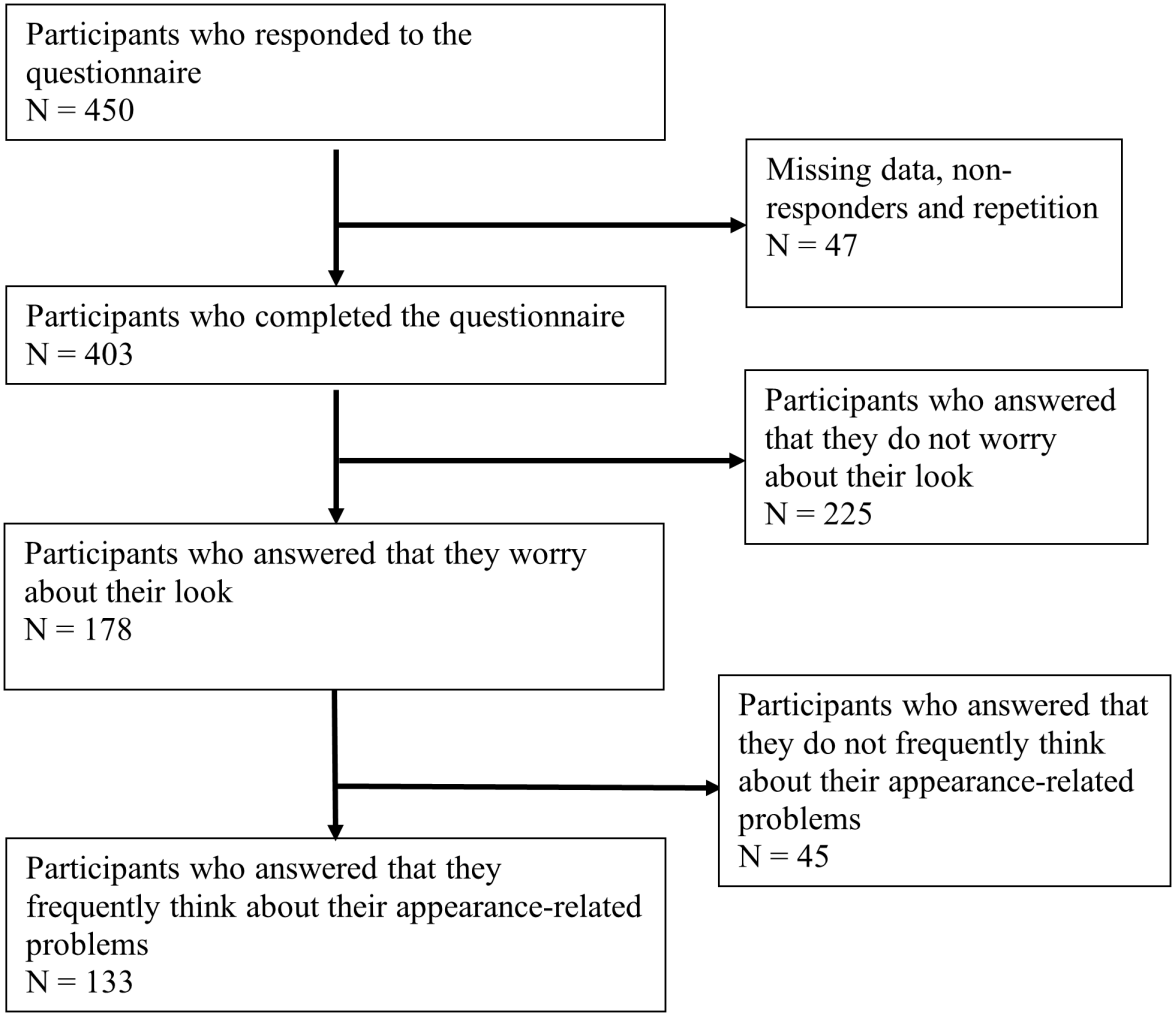
Participants flow chart. Flow chart showing the number of participants included in the study. Participants underwent self-assessment using the BDDQ. The number of participants filtered by each question in the BDDQ are shown.

A total of 403 participants were included in the study (**Figure 1**). The majority were young adults aged 18–35 (84.9%) and of Middle Eastern ethnicity (80.4%) (**Table 1**). Most participants were single (77.7%), with 21.1% married and 1.2% divorced, separated, or widowed. Over half (51.1%) reported an average monthly household income between 2,000 and 29,999 AED, while 34.7% reported incomes above this range and 14.1% reported incomes below 2,000 AED.

**Table 1:** Sociodemographic characteristics and body dysmorphic disorder (BDD) screening outcomes among male participants in the UAE.

Variable	Measure	Number of participants (%)	BDD+ participants	P-Value
Age (Years)	18-35	342 (84.9%)	15	0.25
	36-55	52 (12.9%)	0	
	>55	9 (2.2%)	0	
Ethnicity	Middle Eastern	324 (80.4%)	12	0.48
	White	46 (11.4%)	1	
	Hispanic / Latino	3 (0.7%)	0	
	African American	6 (1.5%)	0	
	Asian / Pacific	19 (4.7%)	1	
	Others	5 (1.2%)	1	
Marital status	Never Married	313 (77.7%)	13	0.67
	Married	85 (21.1%)	2	
	Divorced/Separated/Widowed	5 (1.2%)	0	
Average household monthly income (AED)	<2000	57 (14.1%)	2	0.78
	2000-29999	206 (51.1%)	6	
	30000-49999	85 (21.1%)	4	
	>50000	55 (13.6%)	3	
Height (cm) Mean ± SD	175.4 ± 7.3			0.44^a^
Weight (Kg) Mean ± SD	79.9 ± 17.0			0.03^a^
BMI (Kg/m^2) Mean ± SD	25.6 ± 5.21			0.0099^a^

^a^Mann–Whitney U test applied due to non-normal distribution.

### 3.2 Body dysmorphic disorder questionnaire

Findings obtained from the analysis of 403 participants’ Body Dysmorphic Disorder Questionnaire (BDDQ) are presented in **Supplementary Table 4**. Of these, 178 individuals (44.2%) expressed appearance concerns that met BDD evaluation criteria. Among them, 133 (74.7%) reported frequent appearance-related thoughts. Overall, 53 participants achieved the cutoff for the BDDQ score, with 15 participants screening positive for BDD without weight-related concerns.

### 3.3 Body parts

Body areas disliked by the study participants are shown (in **Table 2**). Overall, 52% of the surveyed participants expressed muscularity as their primary concern, reflecting societal ideals portrayed by the media. In addition, participants identified body shape, facial blemishes, nose shape, and hair concerns as significant areas of worry.

**Table 2:** Body Areas disliked by the study participants.

Body part	Frequency (n)	Percentage of men with worries (%)
Muscularity	69	52%
Body Shape	65	49%
Facial Blemishes	64	48%
Nose	52	39%
Belly	52	39%
Hair	38	29%
Body Hair	35	26%
Chest	30	23%
Face in General	29	22%
Jaw or Chin	25	19%
Asymmetry of a Body Part	22	17%
Skin	21	16%
Skin Color/Texture/Tone	18	14%
Eyes	17	13%
Forehead	16	12%
Hips	15	11%
Feet	13	10%
Ears	10	8%
Hands	5	4%
Others	2	2%

Participants (N = 403) were able to select more than one body part of concern from a general selection of body parts causing body imaging concerns.

### 3.5 Social media and cultural pressure in BDDQ+ men

As predicted, there was a significant association between perceptions of social media’s impact and BDD screening outcomes. Among the 94 participants who reported that social media negatively influenced their feelings about appearance, 10 (10.6%) screened positive for BDD (BDDQ+). In contrast, none of the 189 participants who reported no negative impact screened positive. Of the 120 participants who were uncertain about social media’s effect, 5 (4.2%) screened BDDQ+. A chi-square analysis confirmed this association was statistically significant (χ²(2) = 19.92, p <.001), and Fisher’s Exact Test further supported the result (p = .000), indicating that participants perceiving a negative impact from social media were significantly more likely to screen positive for BDD. Cultural pressure was measured using the specific items described in the Methods section (**Supplementary Table 3**).

Our study evaluated individuals’ subjective perceptions of how social media affected their body image rather than objectively gauging the kind or volume of content they viewed. This subjective metric might not just indicate exposure but also a person’s susceptibility to media influence.

The relationship between feelings of cultural pressure to attain an ideal appearance and positive BDD screening was investigated. Of the 112 participants who reported feeling pressured to conform to a culturally ideal look, 9 (8%) screened positive for BDD (BDDQ+). Among the 190 participants who denied experiencing such pressure, only 3 (1.6%) screened positive. Similarly, of the 101 who were uncertain about experiencing such pressure, 3 (3%) were BDDQ+. These findings suggest that perceived cultural pressure to achieve an ideal look may be associated with a higher likelihood of screening positive for BDD.

### 3.4 Body image and cosmetics in BDDQ+ men

Individuals with BDD were significantly more likely to experience physical discomfort difficulties (OR = 4.9, 95% CI: [1.5, 15.8], p = 0.005). Moreover, a significant association was identified between worry from other people’s perception of body and BDD positivity (OR = 3.9, 95% CI: [1.2, 12.3], p = 0.017). Having underwent cosmetic surgery or any other non-cosmetic appearance modifications was not associated with BDD (**Supplementary Table 5**). However, BDD positive respondents were significantly more likely (OR = 5.8, 95% CI: [2.0, 16.4], p = 0.002) to consider cosmetic surgery.

### 3.6 Exercise in BDDQ+ men

Exercise frequency, wearing multiple layers, using steroids, and going to the gym were not linked to BDD (**Supplementary Table 5**). However, there was a trend toward a higher sense of insufficient muscularity (p=0.066) among males who tested positive for BDD.

### 3.7 Multivariate analysis of factors associated with BDD

Using multivariate logistic regression analysis, three factors were found to be linked to men in the United Arab Emirates who tested positive for BDD (**Supplementary Table 6**). There was an association between higher odds of BDD and reporting particular cosmetic-related body image concerns (OR = 3.85; CI: 1.02 – 15.3, p = 0.048). Social media’s perceived detrimental effects on body image were a powerful independent predictor (OR = 6.02; CI: 2.14 – 22.1, p = 0.002). On the other hand, BDD was negatively correlated with BMI (estimate: -0.173; CI: -0.0142 – -0.332), p = 0.033). Other factors that were not significantly linked to BDD in the final model were steroid use, gym attendance, reported lack of muscle, wearing layers of clothing, exercise frequency, and perceived societal pressure for an ideal appearance. These results demonstrate the distinct contributions of BMI, cosmetic concerns, and social media influence to the risk of BDD in men in the United Arab Emirates.

## 4 Discussion

To our knowledge, this is the first study in the UAE to investigate the patterns and societal risk factors associated with Body Dysmorphic Disorder (BDD) specifically among males. Our findings highlight the significant influence of self-perceptions of social media’s impact on the likelihood of screening positive for BDD. This aligns with a recent study conducted in Saudi Arabia, which found a strong association between BDD and the use of visually driven platforms such as Instagram and Snapchat, with many affected individuals showing a higher propensity toward considering cosmetic surgery (30).

Rapid industrialization and growing exposure to Western beauty standards have had a considerable impact on the United Arab Emirates’ (UAE) changing ideas of masculinity. In many cultures, including the UAE, masculine attractiveness has historically been linked to muscularity and physical power. Male roles in local media, such as television dramas, have traditionally portrayed qualities like leadership and the position of provider as part of the conventional definition of masculinity in the United Arab Emirates (31). These standards have been altered in recent years by global media, especially Western representations, which have raised expectations for male attractiveness. These expectations frequently highlight a lean and toned physique in addition to physical strength, reflecting Western influences on masculinity and body image (32).

Similarly, a study examining the intersection of orbital plastic surgery, BDD, and social media reported an apparent rise in BDD prevalence over the past seven years, which was closely linked to the growing popularity of social media—particularly during the COVID-19 pandemic (33). However, in contrast to previous research (20, 21), our study did not find a significant association between the amount of time spent on social media and BDD symptoms. Instead, our results suggest that the type and tone of social media content encountered may play a more critical role in the development of BDD-related concerns.

Recent studies have shed light on the influence of social media and its correlation with the prevalence of muscle dysmorphia and eating disorders in the Middle East, which may similarly apply to BDD (21, 34). This pervasive influence of social media on individuals’ self-perception and societal norms may contribute to or exacerbate concerns related to body image and BDD (35). The transformative power of social media in the Middle East, as evidenced by various studies, echoes the findings of our study, suggesting that the digital landscape significantly shapes BDD symptoms by reshaping traditional communication channels and societal norms.

While certain body image disorders, such as eating disorders, exhibit lower incidence rates in men compared to women (36), BDD manifests at comparable rates across genders (37). Moreover, it is estimated to affect around 2.2% of male adults and 2.5% of female adults in the United States (10). Concerns regarding muscularity, body shape, and facial blemishes were among the most frequently expressed issues among adult men in the UAE, aligning with findings in the current literature. Notably, in a study comparing common body parts of BDD among men and women, men exhibited heightened concern regarding muscularity, while skin-related issues were prevalent among both genders (38). The observed concern about facial blemishes among the male population in the UAE is consistent with existing literature; for instance, a meta-analysis of dermatology patients indicated that 12.65% of patients exhibited symptoms of body dysmorphic disorder (39), while another study found a 14.1% prevalence rate among Arab dermatology patients (40).

While other studies have shown a higher prevalence of BDD in adolescents and younger adults (13), or higher prevalence in certain ethnicities (41), our study did not find a significant association between the prevalence of BDD among males in the UAE and socio-demographic characteristics, including different age groups and ethnicity. In addition, current marital status and financial circumstances, as measured by monthly household income, showed no association with the prevalence of BDD in our study.

Interestingly multivariate analysis indicated that among men, a lower BMI was independently linked to a higher chance of testing positive for BDD. This result is in line with earlier studies showing that people with lower BMIs may be more dysmorphic and dissatisfied with their bodies, which can have a detrimental effect on their psychological health and quality of life (42, 43). Additionally, it has been determined that body dissatisfaction associated with low BMI is a risk factor for the emergence of eating disorders, which frequently co-occur with anxiety and depression, as well as dysmorphic symptoms (44–46). These findings underline the need for more research on BDD manifestations that are specific to gender and culture, as well as the intricate relationship that exists between BMI, body image, and mental health.

Furthermore, our study identified a significant association between contemplating cosmetic surgery and the presence of BDD in men. This finding resonates with a systematic review that reported a 19.2% prevalence of BDD among individuals seeking cosmetic surgery (47). Indeed, the body area of concern plays a significant role in this context. For example, abdominoplasty candidates have been previously identified as having the highest prevalence of BDD among individuals seeking cosmetic surgery (48). While no direct link was established between altering one’s appearance or undergoing cosmetic surgery and BDD, this may be attributed to the limited number of participants who underwent such procedures. Considering our focus on male participants and the societal stigma surrounding cosmetic surgery, especially within the Arab male community, these outcomes are not unexpected (49). Existing literature suggests that a considerable proportion of individuals seeking cosmetic procedures, ranging from 5% to 15%, meet the diagnostic criteria for BDD (50).

In our study, no association was found between the frequency of exercise or gym attendance, taking steroids, wearing many layers of clothing, or believing that one is not muscular enough, and the development of BDD in the general male population. Nevertheless, the significance of BDD in the context of muscle dysmorphia, and their impact on individuals has been extensively studied. A study conducted in parts of Europe found that 38.5% of gym-goers surveyed were at risk of BDD, with many of them (47.2%) being female (16). Moreover, in a cross-sectional study, the use of performance-enhancing and image-enhancing drugs, such as steroids, was associated with the development of psychopathological disorders including BDD (51). Furthermore, a recent systematic review delved into muscle dysmorphia and its psychological correlations among males in the Middle East, including associations with disordered eating attitudes, perfectionism, and low self-esteem (52). These findings mirror the heightened concerns of insufficient muscularity and body shape among UAE males, suggesting that such psychological traits may underlie or trigger BDD in this population (53). Additionally, recent studies reported a significant prevalence of eating disorders and BDD in Makkah, Saudi Arabia, highlighting the widespread nature of these concerns across the Middle East (54). Their emphasis on the necessity for awareness and intervention to foster healthy eating habits and body perception supports the need for culturally sensitive and comprehensive approaches to address BDD in these populations (55).

Multiple strategies to increase awareness and early diagnosis of BDD have been studied, with psychoeducation involving parental involvement and the use of cognitive behavioral therapy (CBT) being amongst the most successful. As such, increasing self-esteem is thought to be preventive of BDD (56). Applications tailored towards cognitive behavior have also shown promise in BDD prevention (7). For cosmetic surgeries, routine implementation of validated BDD screening instruments has been shown to improve patient care by identifying people who may not benefit from surgical intervention (57). Furthermore, motivational interviewing strategies should ideally be implemented before starting treatment to foster mental health treatment-seeking (58). Ultimately, a comprehensive approach that integrates awareness, screening, and early intervention may significantly improve outcomes for individuals at risk for BDD.

## 5 Limitations

This study has several limitations that should be considered when interpreting the results. First, it utilized self-report measures which could lead to social desirability bias or recall bias. Second, additional validation in this demographic is necessary, even though the BDDQ and other scales were piloted and culturally adjusted. Third, despite a thorough translation procedure, there might be minor interpretational discrepancies between the Arabic and English versions. Due to sample size limitations, no language-specific subgroup analysis was carried out. Fourth, the use of a convenience cross-sectional sample restricts the capacity to draw conclusions about causality and generalizability. Limited statistical power in specific subgroups is reflected in the wide confidence intervals for various odds ratios. Lastly, rather than measuring objective content or usage patterns, the study examined the influence of social media as reported by the participants.

## 6 Conclusion

This study sheds light on mental health and bodily concerns in the general male population in the UAE, particularly addressing the issue of BDD. The findings of this research reveal that perceiving a negative impact of social media, having specific cosmetic-related body image concerns, and lower BMI were independent predictors of BDD. Future research should explore cultural factors and the causal effect of risk factors on BDD. Recommendations include targeted awareness campaigns and community-based resources to address body image concerns in the UAE male community.

## Data Availability

The original contributions presented in the study are included in the article/**Supplementary Material**. Further inquiries can be directed to the corresponding author.

## Supplementary material

Supplementary Table 1 Questions Asked in the BDDQ section. This includes the items included in the BDDQ, a DSM-IV validated questionnaire for screening for BDD. Participants were required to answer yes to the first two questions to continue the rest of the questionnaire.

Supplementary Table 2 Questions Asked in the Body Image and Cosmetics section. This section includes 5 items to determine the relationship between BDD and body image/cosmetics.

Supplementary Table 3 Questions Asked in the Exercise and social media section. This section includes 5 items to determine the relationship between BDD and exercise habits, with emphasis on symptoms/risk factors of muscle dysmorphia. It also includes 3 items to determine the relationship between BDD and social media habits.

Supplementary Table 4 This table details the participants’ responses to the BDDQ section. Overall, participants were required to score 4 on the questionnaire, as well as not having weight as the main concern to screen positive for BDD.

Supplementary Table 5 This table details the participants’ responses to the Body Image & Cosmetics section, as well as the Exercise section.

Supplementary Table 6 Multivariate logistic regression analysis of factors associated with screening positive for body dysmorphic disorder (BDD) among men in the UAE.
