# Supplementary material for: The influence of social media and cultural ideals on body dysmorphic disorder among adult males in the UAE
Source: Front Psychiatry. 2025 Jul 18;16:1613623. doi: 10.3389/fpsyt.2025.1613623 (PMC12313578; doi:10.3389/fpsyt.2025.1613623)
Supplement: Supplementary Table 1 — Questions Asked in the BDDQ section. This includes the items included in the BDDQ, a DSM-IV validated questionnaire for screening for BDD. Participants were required to answer yes to the first two questions to continue the rest of the questionnaire. [file Table1.docx]

**Supplementary Tables**

| BDDQ section |
| --- |
| 1. Are you worried about how you look? |
| 1. Do you think about your appearance problems a lot and wish you could think about them less? |
| 1. Body areas disliked in one's body |
| 1. Is your weight your main concern about how you look? |
| 1. Have your appearance-related insecurities often upset you a lot? |
| 1. Have your appearance-related insecurities often gotten in the way of doing things with friends, dating, your relationships with people, or your social activities? |
| 1. Have your appearance-related insecurities caused you any problems with school, work, or other activities? |
| 1. Are there things you avoid because of how you look? |
| 1. On an average day, how much time do you usually spend thinking about how you look? |

Supplementary Table 1: Questions Asked in the BDDQ section. This includes the items included in the BDDQ, a DSM-IV validated questionnaire for screening for BDD. Participants were required to answer yes to the first two questions to continue the rest of the questionnaire.

| Body Image and Cosmetics section |
| --- |
| 1. Do you feel comfortable with the size and/or shape of your body? |
| 1. Do you worry about how others perceive your body? |
| 1. Have you ever done anything to change your appearance, excluding cosmetic surgery? |
| 1. Did you ever get cosmetic surgery? |
| 1. Have you ever thought about modifying your body by getting cosmetic surgery? |

Supplementary Table 2: Questions Asked in the Body Image and Cosmetics section. This section includes 5 items to determine the relationship between BDD and body image/cosmetics.

| Exercise and social media section |
| --- |
| 1. How often do you exercise? |
| 1. Do you go to the gym? |
| 1. Do you take steroids to increase your muscle mass? |
| 1. Do you believe that you are insufficiently muscular? |
| 1. Do you find yourself wearing multiple layers of clothing to appear “larger”? |
| 1. How much time, on average, do you spend on social media each day? |
| 1. Do you believe that social media has negatively impacted your feelings about your appearance? |
| 1. Have you ever felt pressured to fit into a culturally "ideal" look (ex: strong and muscular for males)? |

Supplementary Table 3: Questions Asked in the Exercise and social media section. This section includes 5 items to determine the relationship between BDD and exercise habits, with emphasis on symptoms/risk factors of muscle dysmorphia. It also includes 3 items to determine the relationship between BDD and social media habits.

| Body dysmorphic disorder questionnaire results | | | |
| --- | --- | --- | --- |
| Criteria | Item | Frequency (n) | Percentage (%) |
| 1. Are you worried about how you look | Yes | 178 | 44.2% |
|  | No | 225 | 55.8% |
| 2. If yes, do you think about your appearance problems a lot and wish you could think about them less? | Yes | 133 | 74.7% |
|  | No | 45 | 25.3% |
| Responded positively to the above two questions | Yes | 133 | 33% |
|  | No | 270 | 67% |
| 3. Is your weight your main concern about how you look? | Yes | 80 | 60.2% |
|  | No | 53 | 39.8% |
| 4. Have your appearance-related insecurities often upset you a lot? | Yes | 88 | 66.2% |
|  | No | 45 | 33.8% |
| 5. Have your appearance-related insecurities often gotten in the way of doing things with friends, dating, relationships, or social activities? | Yes | 92 | 69.2% |
|  | No | 41 | 30.8% |
| 6. Have your appearance-related insecurities caused you any problems with school, work, or other activities? | Yes | 62 | 46.6% |
|  | No | 71 | 53.4% |
| 7. Are there things you avoid because of how you look? | Yes | 102 | 76.7% |
|  | No | 31 | 23.3% |
| 8. On average, How much time do you usually spend thinking about how you look? | Less than 1 hour a day | 79 | 59.4% |
|  | 1-3 hours a day | 47 | 35.3% |
|  | More than 3 hours | 7 | 5.3% |
| BDD scoring (n=403) | 0 | 225 | 55.83% |
|  | 1 | 45 | 11.16% |
|  | 2 | 12 | 2.98% |
|  | 3 | 68 | 16.87% |
|  | 4 | 53 | 13.15% |
| Participants with frequent appearance-related thoughts (n=133) |  | 133 | 100% |
| Participants scored 4 whose weight is the main concern |  | 38 | 28.57% |
| Participants scored 4 whose weight is not the main concern |  | 15 | 11.23% |
| Participants screening positive for BDD |  | 15 | 11.23% |

Supplementary Table 4: This table details the participants’ responses to the BDDQ section. Overall, participants were required to score 4 on the questionnaire, as well as not having weight as the main concern to screen positive for BDD.

| BDD in relation to Questionnaire Sections | | | | |
| --- | --- | --- | --- | --- |
| Social Measure | Item | Responses | BDD+ | P-value |
| Body Image & Cosmetics | | | | |
| Body Comfort | Yes | 253 | 4 | 0.005 |
|  | No | 150 | 11 |  |
| Worry others perception | Yes | 172 | 11 | 0.17 |
|  | No | 231 | 4 |  |
| Done Anything Non-cosmetic to change appearance | Yes | 73 | 3 | 0.741 |
|  | No | 330 | 12 |  |
| Already Underwent Cosmetic Surgery | Yes | 16 | 0 | 1 |
|  | No | 387 | 15 |  |
| Considering Cosmetic Surgery | Yes | 72 | 8 | 0.002 |
|  | No | 331 | 7 |  |
| Exercise & Muscle Dysmorphia | | | | |
| Exercise Habits | Never | 88 | 3 | 0.967 |
|  | Once a week | 93 | 3 |  |
|  | 2-4 times a week | 134 | 5 |  |
|  | Almost Daily | 88 | 4 |  |
| Going to Gym | Yes | 147 | 8 | 0.18 |
|  | No | 256 | 7 |  |
| Steroids Use | Yes | 23 | 0 | 1 |
|  | No | 380 | 15 |  |
| Belief of Being Insufficiently Muscular | Yes | 197 | 11 | 0.066 |
|  | No | 206 | 4 |  |
| Wearing Multiple Layers | Yes | 34 | 1 | 1 |
|  | No | 369 | 14 |  |

Supplementary Table 5: This table details the participants’ responses to the Body Image & Cosmetics section, as well as the Exercise section.
